# The long-acting anticoagulant rodenticide brodifacoum induces neuropathology in adult New Zealand White rabbits and is reduced by the bile sequestrant cholestyramine

**DOI:** 10.1016/j.neuro.2026.103396

**Published:** 2026-01-31

**Authors:** Intakhar Ahmad, Jacqueline Rocha, Zachary McDonald, Douglas L. Feinstein

**Affiliations:** a Department of Anesthesiology, University of Illinois College of Medicine, Chicago, IL 60612, USA; b Research & Development Service, Jesse Brown VA Medical Center, Chicago, IL 60612, USA

**Keywords:** Anti-coagulant, Myelin, Glial cells, Cholestyramine, Brodifacoum

## Abstract

Previous studies showed that exposure to long-acting anticoagulant rodenticides (LAARs) can induce neuropathology in adult rats. In the current study we tested if the potent LAAR brodifacoum similarly promoted neuropathology in adult rabbits which provide a better model of human LAAR poisoning. Adult male New Zealand White rabbits were administered by gavage a single administration of brodifacoum at its LD_50_ dose (200 μg/kg), followed by daily injections of vitamin K1 to prevent mortality due to anti-coagulation. After 3 weeks, examination of the cerebellum revealed an increase in glial cell activation, and a decrease in myelin content. A targeted lipidomics analysis was carried out to determine if brodifacoum altered the relative abundance of lipids enriched in myelin. We observed brodifacoum-dependent decreases in several sulfatides which were associated with an increase in expression of arylsulfatase A which degrades sulfatides. Daily treatment with the bile sequestrant cholestyramine, which accelerates LAAR clearance from the body, ameliorated brodifacoum -induced damage. These findings confirm that, despite daily vitamin K1 treatment, LAARs such as brodifacoum can induce neuropathology in adult animals and support the use of agents such as bile sequestrants to ameliorate those consequences.

## 1. Introduction

Long-acting anticoagulant rodenticides (LAARs) are well characterized for their ability to reduce coagulation via inhibition of vitamin K epoxide reductase (VKORC1) necessary for recycling of vitamin K1 (VK1), required for activity of the enzyme gamma-glutamyl carboxylase (gGC) which carboxylates and activates proteins in the coagulation cascade (Stafford, 2005). LAARs were initially developed as a means of pest control to eradicate rodent populations (Hadler and Shadbolt, 1975). Although the use of LAARs over the past few years has diminished (King and Tran, 2015), they are still used throughout the world and are associated with health risks due to accidental or intentional exposure (Feinstein et al., 2016). Once in the body, LAARs exhibit minimal metabolic breakdown, are strongly hydrophobic, and undergo enterohepatic recirculation; factors which result in extended biological half-lives (Muchiri et al., 2024). While the acute consequences of LAAR poisoning (e.g. reduced coagulation) can be prevented by treatment with fresh plasma or VK1 supplementation, those treatments do not accelerate clearance from the body and therefore do not address potential long-term consequences due to residual stores. To address this, we showed that administration of the FDA-approved bile sequestrant cholestyramine (CSA) reduce LAAR-induced mortality (Lindeblad et al., 2018)and accelerated clearance from the body (Muchiri et al., 2024).

While anti-coagulant actions are responsible for its acute lethality, LAARs also have non-coagulation related effects which could have longterm consequences. VK1 is needed for GGC-dependent carboxylation of other proteins including osteocalcin, involved in energy metabolism and bone remodeling (Li et al., 2016); and the matrix Gla protein involved in decalcification (Schurgers et al., 2013). GGC also carboxylates proteins involved in brain physiology including cerebroside sulfotransferase (CST) needed for myelin synthesis (Eckhardt, 2008). Consistent with this, we reported that warfarin, but not the direct thrombin inhibitor dabigatran reduced brain sulfatide levels in mice (Marangoni et al., 2016a). GGC also activates Gas6 (growth arrest specific gene 6), a ligand of the tyrosine kinase receptors Tyro3, Axl, and Mer (Prieto et al., 2000) which have trophic effects on neurons and oligodendrocytes, and regulate microglial activation (Binder et al., 2008; Grommes et al., 2008; Shankar et al., 2003). Reductions of VK1 levels could therefore influence brain homeostasis as well as coagulation. In addition, LAARs have VK1-independent properties, mediated by direct interaction with cellular membranes. Using X-ray diffraction, we showed that two LAARs, brodifacoum (BDF) and difenacoum (DiF), but not warfarin, intercalate into lipid monolayers altering membrane structure (Marangoni et al., 2016b) which could underlie rapid BDF-induced cell death of neurons (Marangoni et al., 2016b). Treatment with VK1 to restore coagulation will not reduce these VK1-independent actions.

Consistent with the possibility that LAARs could influence activity of CNS proteins, we showed that BDF induced neuropathology in adult rats (Kalinin et al., 2017). By 4 days after administration of BDF, examination of the cerebellum showed increases in astrocyte and microglia activation, as well as an increase in the levels of carbonylated proteins. In subsequent studies we optimized a model of BDF poisoning in adult New Zealand White (NZW) rabbits which better recapitulates events (internal hemorrhage) occurring in poisoned humans (Lindeblad et al., 2018). We therefore hypothesized that BDF would provoke similar changes in adult NZWs. In the current study we show that 3 weeks after administration of BDF there was increased gliosis and decreased myelin staining in the cerebellum. To determine if myelin-enriched lipids were affected by brodifacoum, we carried out a targeted lipidomics analysis for cerebrosides, sulfatides, sphingomyelins, and ceramides. We found alterations in several myelin lipids due to brodifacoum, including a reduction in sulfatide levels. Immunostaining and mRNA analysis revealed an increase in the sulfatide degrading enzyme aryl sulfatase A (ARSA) which could account for reduced sulfatides, while treatment with CSA reduced the consequences of BDF. Together the data demonstrate that LAAR exposure can have long term, neuropathological sequelae that can be minimized by CSA.

## 2. Materials and methods

### 2.1. Chemicals

Cholestyramine (CSA; catalog No. C4650) and brodifacoum (BDF; catalog No. 46036) were obtained from Millipore Sigma (St Louis, Missouri). Vitamin K1 was from Bimeda (Veta-K1, Sueur, Minnesota). A 3.0 mg/mL stock solution of BDF was prepared in 100 % ethyl acetate then diluted in sterile saline to a final concentration of 0.10 mg/mL. CSA was prepared as a 200 mg/mL suspension in sterile saline immediately before use.

### 2.2. Animals and treatments

Two-month old male New Zealand White (NZW) rabbits (n = 8, from Harlan Laboratories) were administered by oral gavage a single bolus of BDF (2 mL/kg of 0.10 mg/mL BDF) to achieve the LD50 dose of 200 μg/kg (Lindeblad et al., 2018). Control rabbits (n = 4) received sterile saline only. To prevent mortality, vitamin K1 (VK1) was administered daily to all rabbits (5 mg/kg s.c.). Beginning 24 h after BDF was administered and 30 min before VK1 was administered, one group (“BDF”, n = 4) of the BDF-treated rabbits were administered 10 mL of saline by oral gavage; and a 2nd group (“BDF&CSA”, n = 4) were administered by oral gavage 10 mL of a 200 mg/mL suspension of the bile sequestrant CSA, which reduces mortality (Lindeblad et al., 2018) and increases BDF clearance (Muchiri et al., 2024), to achieve a daily dose of 0.67 gm CSA/kg. CSA treatment was continued daily for a total of 10 days. Animals were euthanized 21 days after BDF was administered. Euthanasia was accomplished by anesthetization with ketamine (10 mg/kg, intramuscular) followed by an overdose of barbiturate (Euthanasia III solution, 0.4 % pentobarbital sodium, 0.27 mL/kg, administered i.v.). All animal studies were approved by the Institutional Animal Care and Use Committee at the University of Illinois.

### 2.3. Immunostaining

Brains from all rabbits in each group (except for the CSA group which had 3 in the group due to one that was euthanized due to an injury unrelated to the study treatments) were dissected, cut sagittally, then one hemisphere frozen and the other post-fixed in 4 % paraformaldehyde for 2 days. Fixed hemispheres were incubated in increasing sucrose concentrations (10 %, 20 %, 30 %), frozen in OCT and 20 μm sections were prepared and stored in cryoprotective solution (10 % sucrose; 0.01 % sodium azide; pH 7.2 in 160 mM NaP0_4_) until use. Sections were collected onto slides, incubated in blocking solution (2 % normal goat serum and 1 % Triton X-100 in PBS) for 2 h at room temperature, then incubated with primary antibodies overnight at 4°C. The primary antibodies used were rabbit polyclonal anti-GFAP (Abcam #ab68248, 1:300), goat polyclonal Iba1 (Novus Biologicals #NB100–129, 1:300 dilution) and rabbit polyclonal anti-N-terminal peptide of human ARSA (Aviva Systems Biology #ARP54528_P050;1:300 dilution). Sections were rinsed in PBS and incubated with Cy3 or Cy5 conjugated secondary antibodies (1:400; JacksonImmuno Research) for 2 h at room temperature followed by counterstaining of nuclei with DAPI (LifeTechnologies). After washing, coverslips were mounted using mounting medium (Aquamount; Thermo Scientific). Sections were imaged using a Zeiss Axiophot II fluorescent microscope and analyzed using Zeiss Axiovision software. The total area stained (% area stained) was determined in the cerebellar white matter. At least 3 sections per animal and 3 animals per group were used to quantify staining.

### 2.4. Quantitation of cerebellar myelin content

Three cerebellar folia (lobules) were randomly selected from 3 animals per group. Serial 20 μm sagittal sections were matched by selecting a consistent parasagittal hemispheric section. Analyses were restricted to the posterior cerebellar hemisphere, and only sections containing the same anchor fissure, consistent folial geometry, and a comparable arbor vitae branching pattern were included. Planes were further verified by the position of the white-matter core (WM) within the same folial context and, for ARSA, by consistent alignment of WM with the adjacent granule cell layer (GL) and Purkinje cell layer. Sections were stained using Black Gold II (Biosensis, catalog TR-100-BG). Sections on slides were rehydrated in water, incubated in 0.3 % Black-Gold II at 65 °C for 12 min, rinsed with distilled water, then the staining was fixed by immersion in 1 % sodium thiosulfate for 3 min. Slides were cover-slipped and imaged under a 10x objective on a Zeiss microscope with fixed illumination and exposure settings to ensure consistency across samples. For image analysis, a fixed square region of interest (ROI) measuring 4000 pixels (approximately 250 μm × 250 μm) was manually selected following a systematic uniform random sampling and avoiding edges, folds, or other artifacts within the granular layer using a Python-based script. Selected ROIs were processed using a separate Python application which distinguishes myelin-positive regions (black pixels) from myelin-negative regions (white pixels) based on a fixed threshold. The percentages of myelin-positive and myelin-negative pixels and results were exported into Excel for further analysis.

### 2.5. Quantitation of cerebellar ARSA expression

Arylsulfatase A (ARSA) expression in cerebellar white matter was assessed using fluorescence microscopy. Three animals per experimental group were evaluated, with three cerebellar folia (lobules) randomly selected from each animal. Tissue sections were prepared at consistent anatomical planes to minimize variability. Imaging was performed using a Keyence BZ-X800 digital microscope equipped with a Plan Apochromat 20 × BZ-PA20 objective, under standardized illumination, exposure, and imaging settings to ensure linear, unsaturated pixel intensity values. A square region of interest (ROI) approximately 110 μm × 110 μm was randomly selected using systematic uniform random sampling within the cerebellar white matter of each folium. ROIs were processed using an OpenCV-based Python application to obtain total fluorescence intensity, defined as the sum of pixel intensities within each ROI, normalized to blank samples to correct for background fluorescence.

### 2.6. RNA analysis

Frozen cerebelli from 3 animals per group were homogenized in RNAzol RT (Molecular Research Center, cat #RN190) and RNA precipitated according to the manufacturer’s protocol. One μg total RNA was converted to cDNA using the High Capacity cDNA Reverse Transcription Kit (Applied Biosystems, cat #4368814). cDNA samples were amplified using primers

ARSA forward 5’-TTGGATGGCTTTGACCTCAG and

ARSA reverse: 5’-CTTGTACTTCCCACTCCGAAC

for arylsulfatase A (ARSA) with SsoAdvanced Universal SYBR Green Supermix (BioRad, cat #1725272) in a BioRad CFX96 real time PCR machine (BioRad, Hercules, CA). Relative mRNA levels were calculated from Ct values and normalized to β-actin mRNA levels in the same samples using primers

Actin forward 5’-CAAGCAGGAGTATGACGAGTC and

Actin reverse 5’-AAACACGAATAAAGCCATGCC

### 2.7. Lipid analysis

Multidimensional mass spectrometry-based targeted lipidomics was carried out at the University of Texas Health San Antonio Functional Lipidomics Core directed by Dr Xianlin Han, as previously described (Palavicini et al., 2016). Briefly, pulverized frozen cerebelli from 3 rabbits per group (naïve, BDF only, and BDF plus CSA treated) were homogenized in ice-cold PBS using a Precellys^®^ Evolution Tissue Homogenizer (Bertin, France). The protein concentration of homogenates was determined using Pierce BCA Protein assay (Thermo Fisher, USA, Cat# 23225). Lipids were extracted by the modified procedure (Cheng et al., 2007) of Bligh and Dyer (1959) in the presence of internal standards added based on the total protein content of each sample. Lipids were quantified by ion peak intensity comparison to the internal standard of the class of lipids, acquired on a triple-quadrupole mass spectrometer (TSQ Altis, Thermo Fisher Scientific, Waltham, MA, USA) equipped with a TriVersa NanoMate^®^ device (Advion Interchim Scientific, USA) on an Xcalibur operating system as described (Wang et al., 2017). Data processing including ion peak selection, baseline correction, data transfer, peak intensity comparison, and quantitation was performed as described (Cheng et al., 2007; Qiu et al., 2021). Results were normalized to total protein content to give nmol lipid/mg protein. Additional details of sample preparation and mass spectrometry analysis are provided as Supplemental Data 1.

### 2.8. Data and statistical analysis

**Fig. 1.**
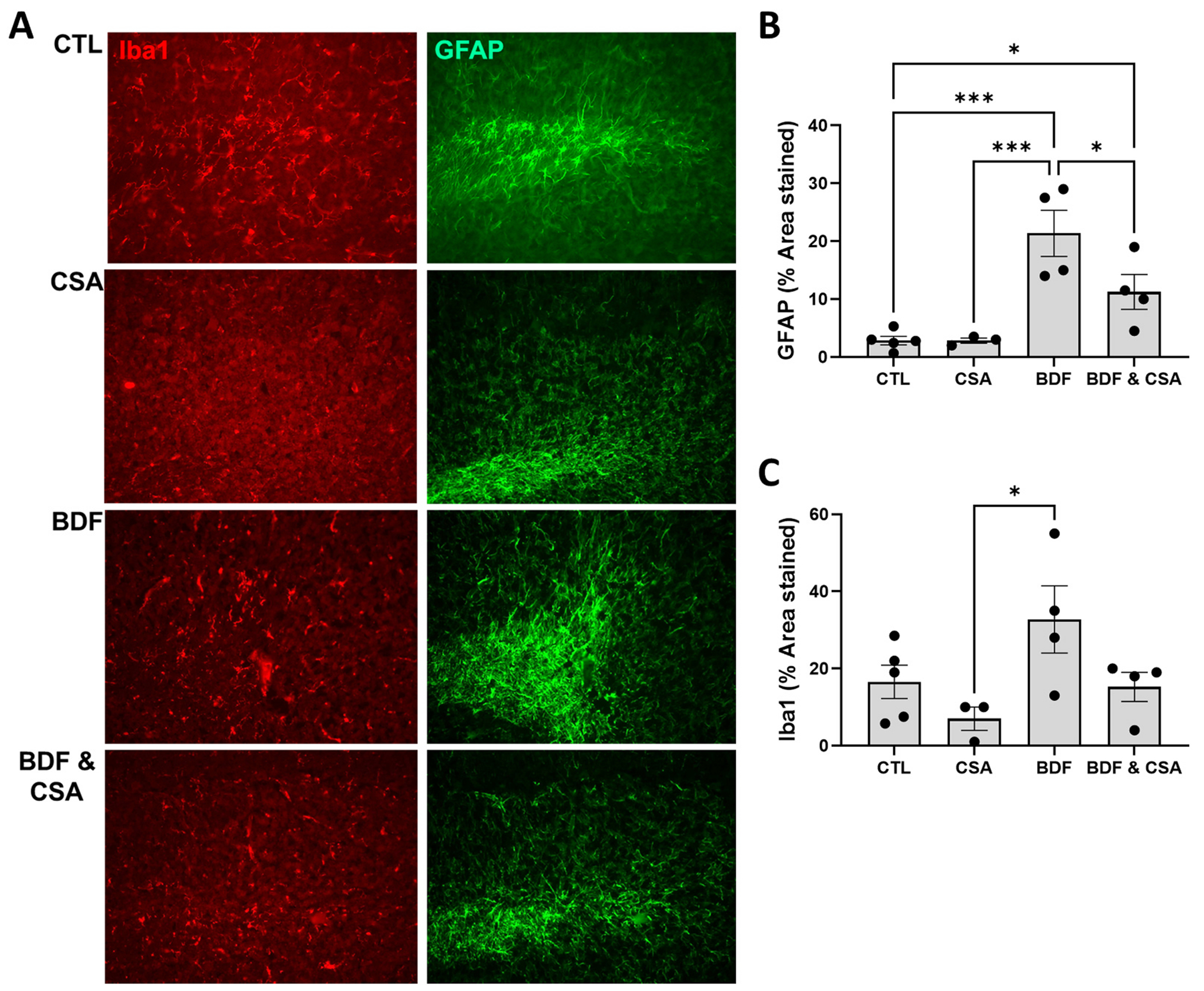
BDF induces cerebellar glial cell activation. (A) Representative images showing GFAP (green, astrocytes) and Iba1 (red, microglial) staining in cerebellar sections from control (CTL), BDF-treated (BDF), CSA only treated (CSA), and BDF and CSA treated (BDF&CSA) 3 weeks after administration of BDF. (B) Quantitation of GFAP staining. Ordinary 1-way ANOVA, Fisher’s post hoc comparison. (C) Quantitation of Iba1 staining. Kruskal-Wallis 1-way ANOVA, Dunn’s post hoc comparisons. Data is mean ± sem, n = 3 or 4 per group. *, p < 0.05; *** p < 0.0005. VK1 was given to all groups.

**Fig. 2.**
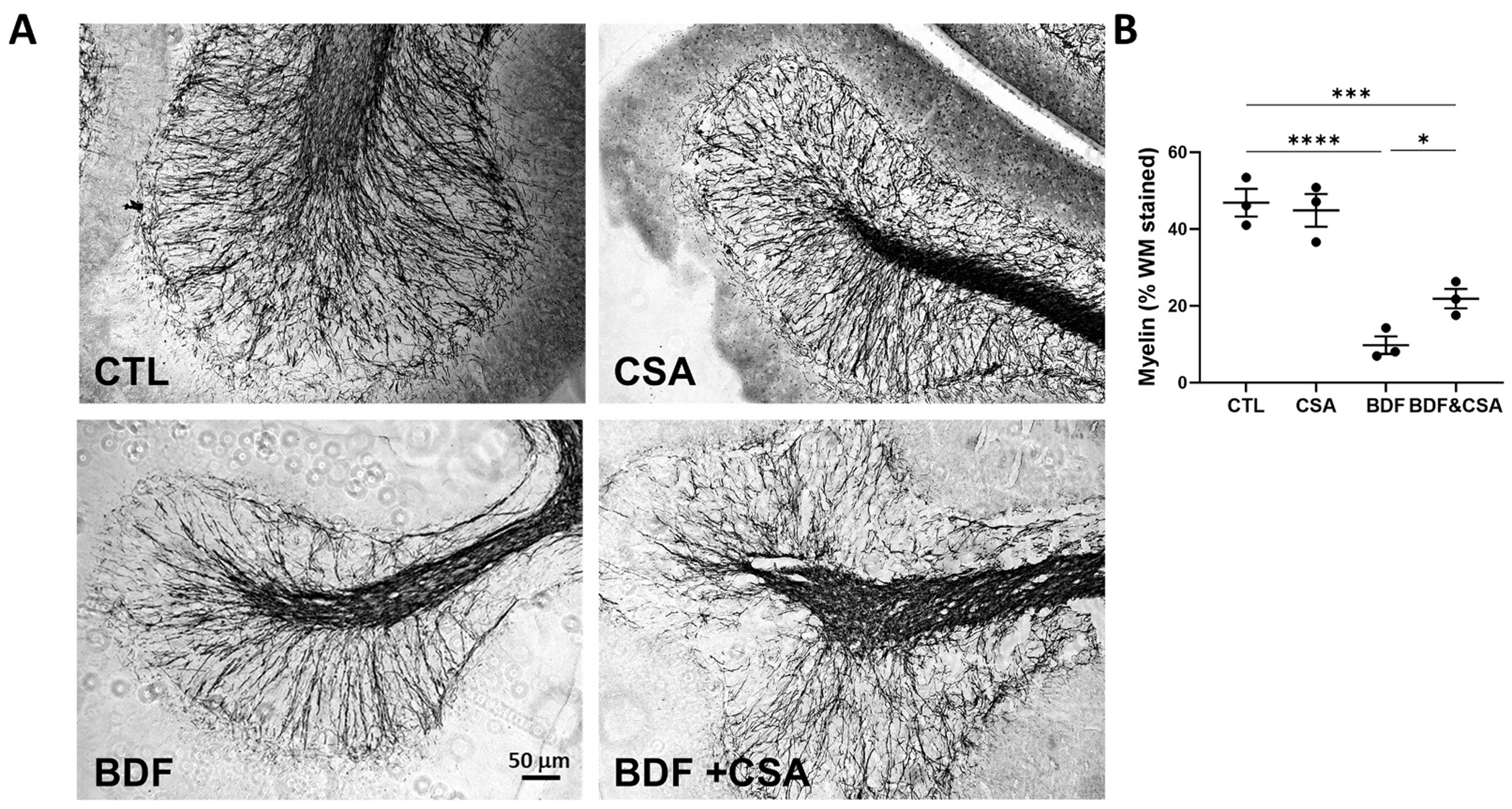
BDF induces cerebellar myelin loss. (A) Representative bright field images showing Black Gold II staining in cerebellar sections from control (CTL), BDF-treated (BDF), CSA only treated (CSA), and BDF and CSA treated (BDF&CSA), 3 weeks after administration of BDF. (B) Quantitation of Black Gold II staining. Data is mean ± sem of the % area stained from 3 sections per rabbit, n = 3 rabbits per group. *, p < 0.05; ***, p < 0.005; ****, p < 0.0005; Ordinary 1-way ANOVA, Fisher’s post hoc comparison. VK1 was given to all groups.

**Fig. 3.**
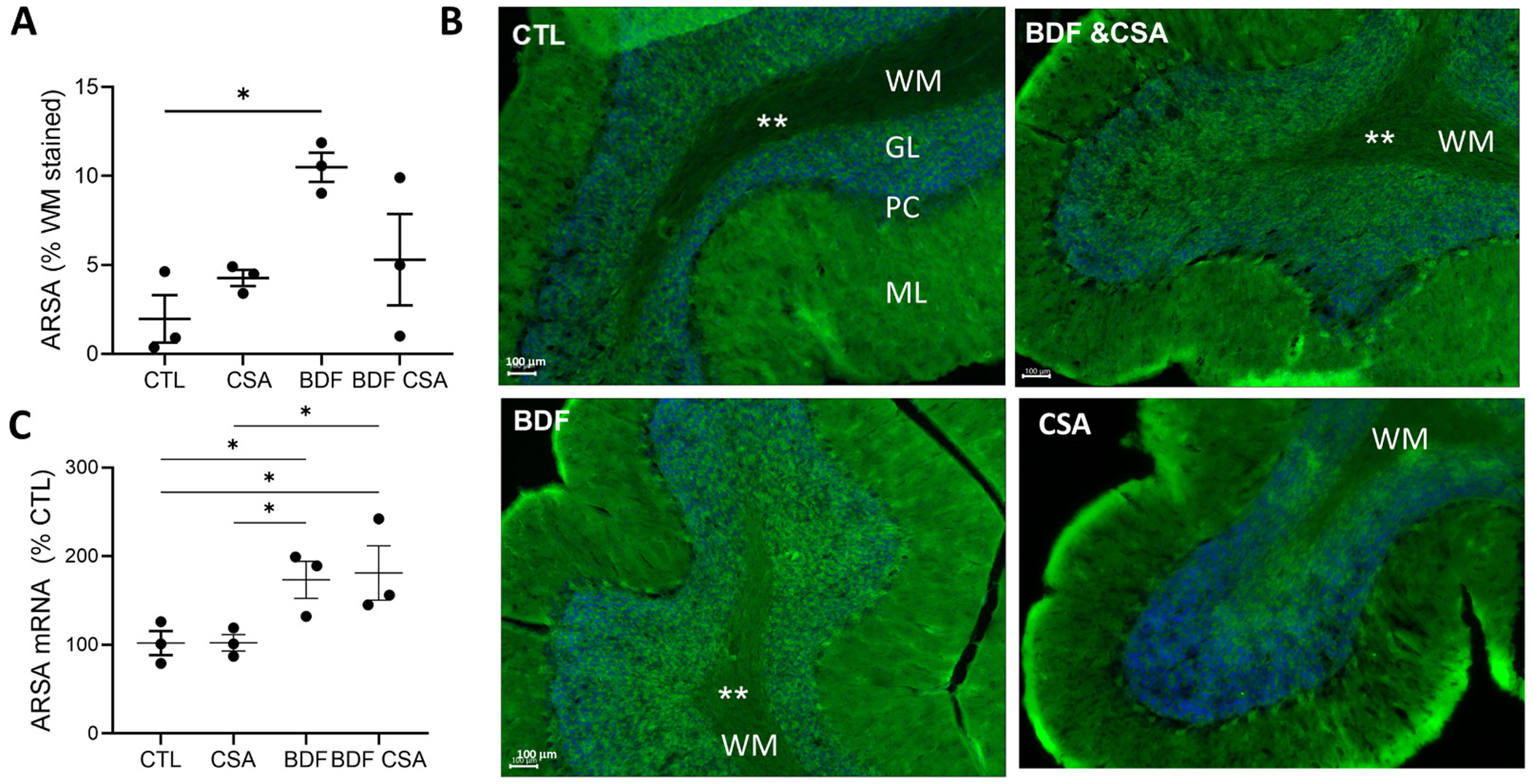
BDF increases cerebellar Arylsulfatase A (ARSA) expression. (A) qPCR of n = 3 samples per group (n = 2 for BDF&CSA) shows an increase in relative ARSA mRNA levels in the cerebellum of BDF treated rabbits compared to samples isolated from naïve or CSA alone treated animals. Data is mean ± sem of 3 sample per group. Ordinary 1-way ANOVA, Fishers post hoc comparison, **, p < 0.005. (B) Immunostaining of shows that BDF also increased ARSA protein expression in cerebellar white matter (WM), which was slightly reduced by treatment with CSA. Granule layer (GL), and Purkinje cells (Arrows). Data is mean ± sem of 3 sections per rabbit, from 3 rabbits per group. Ordinary 1-way ANOVA, Fishers post hoc comparison, *, p < 0.05. VK1 was given to all groups.

All studies (immunostaining, histochemical staining, lipid analyses) were carried out in a blinded fashion. All analyses were performed on individual brain samples. For GFAP and Iba1 immunostaining (Fig. 1), there were 4 rabbits per group except for CSA with 3 rabbits. For myelin staining (Fig. 2), ARSA staining (Fig. 3A), and ARSA qPCR (Fig. 3B) there were 3 rabbits per group. For lipid analyses there were 3 rabbits per group. For immunostaining, the mean % area stained from 3 serial sagittal sections per rabbit through the cerebellum was used for statistical comparisons. Data sets were tested for normality using the Shapiro-Wilk test and all found to show a normal distribution except for the immunofluorescent data for Iba1 shown in Fig. 1C. To evaluate combined effects of BDF and CSA treatment, 2-way ANOVA analysis was performed; however, there were no significant interactions detected between these two factors, the data were therefore analyzed by 1-way ANOVA with Fisher’s least significant difference test to identify differences between groups. In all cases the ANOVA test showed a significant effect (P < 0.05) across groups. For Iba1 groups were compared using the non-parametric Kruskal-Wallis 1-way ANOVA followed by Dunn’s post hoc tests. The ANOVA test did not show a significant difference across all groups (P = 0.103). However post hoc tests identified a significant (P < 0.05) difference between CSA and BDF groups, likely due to the increased power post-hoc tests have to detect differences between 2 groups versus the ANOVA which averages differences across all groups. Results of lipid analyses for each group (naïve, BDF, BDF&CSA) were normalized to 100 % for each of 4 classes (sphingomyelins; ceramides; sulfatides; cerebrosides), then groups compared pairwise by *t*-tests. Unless otherwise indicated, at least 3 independent biological replicates were carried out for each experiment, outliers were identified using Grubb’s test and are indicated in Table 2 by an asterisk. P-values < 0.05 were considered statistically significant. All statistical analyses were carried out using GraphPad Prism 10.6 (GraphPad Software, San Diego, CA).

## 3. Results

### 3.1. BDF exposure increases cerebellar neuropathology

Based on our previous studies which showed that BDF induces neuropathology in adult rats we tested effects of BDF in adult New Zealand Rabbits (NZWs). Immunostaining of the cerebellum done 21 days after BDF was administered (Fig. 1A) shows BDF significantly increased astrocyte activation (GFAP staining) compared to samples from controls (CTL) rabbits approximately 8-fold (Fig. 1B). In contrast BDF increased microglial activation (Iba1 staining) by approximately 50 % (Fig. 1C) however that increase did not reach statistical significance. The increase in GFAP staining was significantly reduced by treatment with CSA, which alone did not alter GFAP staining. Although Iba1 staining due to BDF was decreased by CSA, that decrease was not significant. However, the reduction of Iba1 staining due to CSA alone was significantly different from that observed in the BDF samples. Together these data point to an increase in glial cell activation due to BDF which is lessened by co-treatment with CSA.

### 3.2. BDF reduces cerebellar myelin content

Increased glial activation in the cerebellar white matter suggested possible effects of BDF on myelin content, and staining with Black Gold-II revealed a significant reduction of myelin in cerebellar white matter (Fig. 2A). Quantitation (Fig. 2B) confirmed a 4-fold reduction of staining for myelin in BDF-treated sections compared to CTL sections, and that reduction was partially prevented by treatment with CSA (which alone had no effect on myelin staining).

### 3.3. Targeted lipidomics identifies BDF-induced alterations in myelin enriched lipids

By inhibiting VK1 oxidoreductase component 1 (VKORC1), BDF reduces VK1 levels. Since VK1 is a necessary cofactor for the enzyme CST (cerebroside sulfotransferase, which converts cerebrosides to sulfatides), we hypothesized that BDF led to changes in cerebellar lipid abundance. Targeted lipidomics was used to quantify cerebellar levels of four lipid classes that are enriched in myelin: cerebrosides; sphingolipids; ceramides; and sulfatides. We compared the lipid profiles of cerebellar samples prepared from control, BDF-treated, and BDF&CSA-treated adult rabbits (**full list in** Supplementary Table 1). The total lipids in each class (Table 1) were similar between the 3 groups; and the ratio of cerebrosides to sulfatides was 3.7 in the naïve samples and was not altered by BDF (or BDF &CSA). We found that 5 lipids (Table 2) were significantly (p < 0.05, paired *t*-test; BDF versus naïve) altered by BDF (3 decreased, 2 increased) and treatment with CSA reduced changes in 4 of them (all but sulfatide 12:0). BDF also led to changes in 2 cerebrosides (N24:0 and OH_N24:0) although those changes did not reach statistical significance.

### 3.4. Reduced sulfatide levels are associated with increased expression of arylsulfatase A

The reductions observed in sulfatides could be due to an increase in sulfatide-degrading enzymes such as arylsulfatase A (ARSA). Immunostaining (Fig. 3A) showed an almost 5-fold increase of ARSA staining in cerebellar white matter, which was slightly less in the BDF&CSA samples (Fig. 3B). The increase in ARSA immunostaining was paralleled by a significant increase in relative ARSA mRNA levels (Fig. 3A), which was partially reduced by CSA.

## 4. Discussion

In the current study we show that exposure to a single administration of BDF leads to observable increases in glial cell activation, reductions in myelin content and sulfatide levels, and an increase in ARSA expression when measured in the cerebellum 3 weeks later. These results are similar to our previous observations of BDF effects in adult rats (Kalinin et al., 2017). In that study, BDF increased both astrocyte and microglial activation measured 4 days after BDF was administered. That those effects involved reduction of CNS VK levels is supported by observations showing an increase of GFAP and Iba1 staining in adult mice after 4 weeks treatment with warfarin (Marangoni et al., 2016a); but not in mice administered the direct thrombin inhibitor dabigatran. Although the effects of BDF and other LAARs on neuroinflammation are limited, several studies have shown that glial physiology is influenced by changes in VK. It is well established that VK has numerous functions in the CNS (Ferland, 2012); A. (Popescu and German, 2021), including improving metabolism, reducing cell death, and suppressing of inflammation, and perturbations in VK levels have been associated with increased risk of neurological diseases including Alzheimer’s disease (Annweiler et al., 2015; Carrie et al., 2011; Chouet et al., 2015; Ferland et al., 2016). Direct effects of VK on glial cells have been demonstrated in vitro, for example incubation with VK2 reduced activation of microglial cells by LPS (Saputra et al., 2019) or by rotenone (Yu et al., 2016); and prevented oxidative stress in oligodendrocytes (Li et al., 2009). In rats VK2 ameliorated the development of experimental autoimmune encephalomyelitis, a model of multiple sclerosis, and was associated with reduced glial activation (Moriya et al., 2005). In mice provided a low VK diet had reduced numbers of newly generated neurons, and an increase in activated microglia in the hippocampus (Zheng et al., 2025). The mechanisms by which VK2 exerts beneficial effects are not well defined, but may be due in part to its ability to transport electrons (Lovern and Marbois, 2013) thereby increasing mitochondrial function.

While the anti-coagulant actions of LAARs are well known and well characterized, there are limited studies which have examined their non-anticoagulant effects. In addition to activation of coagulation factors, GGC can carboxylate and activate a variety of other proteins throughout the body, including several in the CNS such as Gas6 (growth arrest specific gene 6, a ligand for the TAM family of tyrosine kinase receptors). Gas6 is widely expressed throughout the CNS (Prieto et al., 1999), and provides trophic support for neurons and oligodendrocytes (Binder et al., 2008; Funakoshi et al., 2002; Shankar et al., 2003). Gas6 also binds to the Tyro3 receptor on microglial to regulate inflammatory activation (Grommes et al., 2008; Prieto et al., 1999; Shankar et al., 2003). In adult rats, treatment with warfarin reduced levels of brain Gas6 carboxylation, as well as in primary astrocytes and microglial cells (Aydin et al., 2024). Warfarin was also shown to reduce VK2 levels in rodents (Tamadon-Nejad et al., 2018) and VK2 levels were lower in human autopsy brain samples from warfarin-treated patients (Shea et al., 2025). It has also been demonstrated that VK2 can suppress microglial activation via inhibition of NFkB (Saputra et al., 2019). Together these studies suggest numerous ways by which reduced VK could lead to increased glial cell activation.

We observed a decrease in myelin staining in the cerebellar white matter, and reduced levels of 2 sulfatides. ARSA removes the sulfate group from sulfatides converting them to galactosylceramides; our findings of increased ARSA expression and activity could therefore account for reduced sulfatide levels. Consistent with this, in the same samples we detected a significant increase in ceramide 18:0 concentration. While changes in other sulfatide metabolic or catabolic enzymes could also contribute to these findings, qPCR analysis of a panel of relevant mRNAs did not reveal any significant differences other than for ARSA (data not shown). It is also possible that reduced VK levels could cause a reduction in CST activity, a VK-dependent protein which requires carboxylation for full activity (Sundaram and Lev, 1990; Sundaram et al., 1996). In our previous studies we found that warfarin, but not dabigatran, in addition to increasing glial inflammation also reduced sulfatide levels (Marangoni et al., 2016a). Similarly, young mice treated with warfarin for 2 weeks showed a dramatic decrease (42 %) in brain sulfatides (Sundaram and Lev, 1988; Sundaram et al., 1996) shown to be due to reduced activity of CST (Sundaram and Lev, 1990). In contrast, in adult rats there are positive correlations between hippocampal sulfatide and VK2 levels (Crivello et al., 2010); and in a model of chemically induced demyelination, administration of VK increased the production of total brain sulfatides during remyelination (Popescu et al., 2018). The basis for increased ARSA expression is unknown, but could be a consequence of BDF-induced stress, for example by causing mitochondrial dysfunction, oxidative stress, or microglial activation, as part of a homeostatic response to restore sphingolipid equilibrium and preserve myelin integrity (Tamadon-Nejad et al., 2018). Increased ceramide accumulation in mitochondria can impair electron transport chain function, leading to increased reactive oxygen species production and neuroinflammation, which can further exacerbate demyelination (Diaz-Vegas et al., 2023; Vos et al., 2021). The effects of BDF of ARSA, sulfatides, and myelin integrity may therefore represent the culmination of Multiple, interacting events.

The above considerations suggest that BDF-dependent reductions in CNS VK levels could account for increased neuroinflammation and reduced sulfatides. However, in the current study the rabbits are given daily VK1 (5 mg/kg s.c.) to prevent hemorrhage and death, therefore reduced CNS levels are not, a priori, expected. This can be explained since the major form of VK in the CNS is menaquinone-4, a prenylated form of VK3 which in contrast to VK1 can pass through the blood brain barrier. Conversion from VK1 to menaquinone (MK)-4 takes hours to days (Yamamoto et al., 1997) with brain accumulation reaching highest levels after several days (Al Rajabi et al., 2012). Therefore, it is likely that in our studies, CNS levels of MK-4, required for activation of CNS VK-dependent proteins, are reduced at least during the first several days following BDF administration. In addition, sulfatide depletion can activate both microglial cells and astrocytes (Qiu et al., 2021) suggesting that an initial decrease in sulfatide content, due to increased ARSA or to a reduction in activation of CST, could initiate glial cell activation which in turn could promote further myelin damage.

In addition to non-coagulant, VK1-dependent actions, LAARs can also exert VK1-independent effects. Addition of BDF or DIF, but not warfarin, to primary cell cultures showed an increase in neuronal cell death and reduced metabolic function; while in astrocytes increased cell damage and death only occurred following depletion of membrane cholesterol (Marangoni et al., 2016b). X-ray diffraction studies showed that BDF and DIF can intercalate into artificial monolayers composed of dipalmitoyl phosphatidylcholine, and lead to disruption of lipid organization (Marangoni et al., 2016b). Molecular dynamics simulations predict that BDF partitions into lipid bilayers, causes membrane thinning, promotes lipid exchange between inner and outer membranes, and can induce formation of water pores which could allow increased water accumulation and cell death (Ayee et al., 2016). More recently we showed that BDF exposure altered the cecal microbiome in adult rabbits (Naqib et al., 2025), leading to significant changes in relative abundance of several bacterial species. Those changes predicted an effect on arginine metabolism, which we confirmed by showing reduced serum concentrations of arginine as well as increased concentrations of nitrite. The latter is likely due to activation of peripheral inflammatory responses including induction of nitric oxide synthase 2 expression in macrophages, which suggests effects of NO on vasodilation. We also showed that BDF significantly alters the numbers and size distribution of extracellular vesicles (EVs), that BDF is found associated with EVs, and that EVs from BDF-treated rabbits can induce cell death in primary microglial cultures (Ahmad et al., 2025).

Further analysis shows that the N24:1 / N24:0 ratio was increased by BDF in rabbits both for cerebrosides (from 1.08 to 1.16) and sulfatides (from 1.84 to 2.34); in both cases this was primarily due to a reduction in levels of N24:0; and in both that reduction was reversed by treatment with CSA (for cerebrosides back to 1.09; for sulfatides to 1.68). The selective loss of the N24:0 species suggests substrate selectivity of lipid breakdown, which is supported by studies showing that saturated sulfatides accumulate to a greater extent than unsaturated sulfatides in human iPSCs (Frati et al., 2018). Unsaturated lipids increase membrane fluidity compared to saturated lipids due to the presence of the double-bond which introduces a kink into the molecule (Harayama and Antonny, 2023; Los and Murata, 2004). This can lead to changes in membrane packing; myelin compaction; thinner or looser sheaths; and eventually pathology (Malone and Szoke, 1982; Morini and Pedroni, 2025; O’Brien, 1965). An increase in the N24:1/N24:0 ratio could therefore contribute to the observed loss of myelin due to BDF.

Limitations to this study include that only a single time point (3 weeks after BDF was administered) was examined and therefore our findings may under- or over-estimate changes that occurred at earlier times. We did not directly measure VK levels in tissues, nor did we measure levels of VK metabolites in the CNS to confirm that VK2 levels remained low despite daily VK1 treatment. We only assessed effects of BDF on glial cell activation using two canonical markers (GFAP and Iba1); it is likely that other markers of glial activation are influenced by BDF. Nor did we carry out characterization of neuronal pathology; which could be directly disturbed by BDF or changes in VK1, or as a secondary consequence of glial cell activation. Additionally, brains were prepared by immersion fixation rather than by perfusion with fixative which could introduce artefacts into assessment of morphological parameters. Finally, in cases of human BDF poisoning, treatments that restore coagulation are sufficient to allow patients to be released from emergency rooms; there are few, if any studies that have examined long-term consequences of BDF poisoning on neuropathology or neurological function; therefore it remains unknown if similar glial cell activation occurs in those cases. Regardless, our data suggests that treatments to accelerated clearance of BDF from the body may provide an additional means to minimize potential long-term consequences.

## 5. Conclusion

The current findings demonstrate that a single exposure to the potent LAAR BDF can have long-term neuropathological consequences. The variety of processes and pathways in the CNS which can be influenced by perturbations in VK levels underscores the potential for long-term damage; while the existence of VK-independent actions of LAARs emphasizes the fact that VK supplementation may not be sufficient to prevent those consequences. Treatment of cases of human LAAR poisoning which accelerate clearance from the body, such as bile sequestrants as used here, could therefore be considered for patient care as well.

## Supplementary Material

1

2

## Figures and Tables

**Table 1:** Total Lipids in each class.

Class	Control	BDF	BDF & CSA
**CBS**	35.47 (9.75)	28.14 (21.2)	30.73 (23.4)
**Sph**	17.10 (0.54)	16.08 (3.19)	16.66 (1.56)
**Cer**	1.28 (0.10)	1.16 (0.22)	0.98 (0.07)
**Sulf**	9.58 (2.43)	7.43 (3.59)	7.36 (3.80)
**Total**	63.43 (12.78)	52.83 (27.80)	55.75 (28.69)
**CBS:Sulf**	3.70	3.79	4.17

Data is mean ± SD, nmoles/mg protein measured in cerebellar samples from 3 rabbits in each group. CBS, cerebrosides; Sph, sphingomyelins; Cer, ceramides; Sulf, sulfatides.

**Table 2:** Lipids significantly altered by BDF.

Class	Lipid	Control	BDF	BDF & CSA
**Sphingomyelin**	**N18:0**	0.546 (0.059)^a^	0.570 (0.073)^b^	0.535 (0.093)
	**N24:1**	0.120 (0.039)^a^	0.108 (0.058)^b^	0.176 (0.033)*
**Sulfatide**	**N12:0**	0.094 (0.030)^a^	0.066 (0.005)*	0.036 (0.015)*
	**N24:0**	0.156 (0.017)^a^	0.129 (0.039)^d^	0.189 (0.036)*
**Ceramide**	**N18:0**	0.824 (0.038)^a^	0.853 (0.025)^b^	0.813 (0.023)
**Cerebroside**	**N24:0**	0.161 (0.012)^c^	0.141 (0.033)	0.143 (0.043)
	**OH_N24:0**	0.166 (0.011)^c^	0.185 (0.036)	0.179 (0.024)

Data is the mean ± SD of the relative expression (%) of indicated lipid in each class, measured in cerebellar samples from 3 rabbits in each group;

* indicates an outlier was identified leaving 2 per group.

a, p < 0.05 vs Naïve

b, p < 0.05 vs BDF

c, p < 0.10 vs Naïve

d, p *<* 0.10 vs BDF.

## Data Availability

Data will be made available on request.

## Appendix A. Supporting information

Supplementary data associated with this article can be found in the online version at doi:10.1016/j.neuro.2026.103396.
