# Supplementary material for: The long-acting anticoagulant rodenticide brodifacoum induces neuropathology in adult New Zealand White rabbits and is reduced by the bile sequestrant cholestyramine
Source: Neurotoxicology. Author manuscript; Available in PMC 2026 Jul 11. (PMC13355867; doi:10.1016/j.neuro.2026.103396)
Supplement: 1 [file NIHMS2190215-supplement-1.docx]

**Lipid extraction and mass spectrometric analysis of lipids**

Multidimensional mass spectrometry-based shotgun lipidomics was performed as previously described (1). Briefly, pulverized frozen CRM and SC tissues were homogenized in ice-cold phosphate-buffered saline using a Precellys® Evolution Tissue Homogenizer (Bertin, France). The protein concentration of homogenates was separately determined with a Pierce BCA Protein assay (Thermo Fisher, USA, Cat# 23225) according to the manufacturer’s instructions. Lipids were extracted by the modified procedure of Bligh and Dyer extraction in the presence of internal standards that were added based on the total protein content of each sample (2). Lipids were quantified by ion peak intensity comparison to the internal standard of the class of lipids as acquired by a triple-quadrupole mass spectrometer (TSQ Altis, Thermo Fisher Scientific, Waltham, MA, USA) equipped with a TriVersa NanoMate® device (Advion Interchim Scientific, USA) on an Xcalibur operating system as previously described. Data processing including ion peak selection, baseline correction, data transfer, peak intensity comparison and quantitation was performed as previously described (3, 4). The results were normalized to the protein content (nmol lipid/mg protein).

1. Palavicini JP, Wang C, Chen L, Ahmar S, Higuera JD, Dupree JL, Han X. Novel molecular insights into the critical role of sulfatide in myelin maintenance/function. J Neurochem. 2016;139(1):40-54.

3. Qiu S, Palavicini JP, Wang J, Gonzalez NS, He S, Dustin E, et al. Adult-onset CNS myelin sulfatide deficiency is sufficient to cause Alzheimer's disease-like neuroinflammation and cognitive impairment. Mol Neurodegener. 2021;16(1):64.

2. Cheng H, Jiang X, Han X. Alterations in lipid homeostasis of mouse dorsal root ganglia induced by apolipoprotein E deficiency: a shotgun lipidomics study. J Neurochem. 782 2007;101(1):57-76. 783

4. Wang M, Wang C, Han X. Selection of internal standards for accurate quantification of complex lipid species in biological extracts by electrospray ionization mass spectrometry-What, how and why? Mass Spectrom Rev. 2017;36(6):693-714.
